# Parasites, Zoonoses and War: A Themed Issue in Honor of Emeritus Professor John M. Goldsmid

**DOI:** 10.3390/tropicalmed5020103

**Published:** 2020-06-21

**Authors:** Richard S. Bradbury

**Affiliations:** School of Health and Life Sciences, Federation University, Berwick 3806, VIC, Australia; r.bradbury@federation.edu.au; Tel.: +61-3-5327-6584

This Special Issue of *Tropical Medicine and Infectious Disease* is dedicated to the life and work of Emeritus Professor John Marsden Goldsmid. Herein, Prof. Goldsmid’s colleagues have contributed papers celebrating his academic contribution to the field of parasitology and zoonosis. Prof. Goldsmid’s outstanding contributions to medical parasitology, the Australasian College of Tropical Medicine and other learned societies are well known in Australia and elsewhere. He is held in justifiable high esteem. Further to this, Prof. Goldsmid was a gifted teacher and enthusiastic mentor to all those who he touched in his decades working at the University of Tasmania. Prof. Goldsmid’s career has included work in the field of parasitology to benefit human and animal health, in peacetime and in war, in both Africa and Australia. 

This Special Issue contains a total of nine original, peer-reviewed papers, many of them published by Prof. Goldsmid’s former colleagues and co-researchers. Included is a paper co-authored by Prof. Goldsmid himself with Dr. Silvana Bettiol, reflecting on the changes in global medicine, parasites, and Tasmania over the 50 years spanning his career [1]. Further reflection on a lifetime of practice is provided by Prof. Ahmed Latif, a former colleague of Prof. Goldsmid, who summarizes his experience on the importance of cultural understanding in the delivery of healthcare while working as a medical practitioner in Africa and remote Australian Aboriginal communities [2].

Prof Goldsmid focused on human and wildlife diseases in Tasmania in the latter years of his career, often in conjunction with Dr. Silvana Bettiol. In keeping with this, Dr. Bettiol, Dr. Bruce Lyons and Emeritus Prof. Greg Woods from the University of Tasmania have contributed a thought-provoking review of Tasmanian devil facial tumour disease (DFTD). This review takes the novel and engaging approach of comparing aspects of the transmissibility and life cycle of DFTD with that of parasitic organisms and, in so doing, provides an easily accessible analogy for the understanding of the epidemiology of this disease of an iconic Tasmanian marsupial [3]. 

Reflecting the major focus and impact of Prof. Goldsmid’s life and work, the majority of the papers in this issue focus on parasitic diseases. A review of human infections with the false hookworm, *Ternidens deminutus*, described work on the epidemiology, clinical manifestations, pathology, diagnosis and treatment of this neglected helminthic disease, much of it summarizing seminal work performed by Prof. Goldsmid himself in Zimbabwe during the 1960’s and 1970’s [4]. 

Prof. Goldsmid promoted the field of travel medicine in its infancy. This theme and that of African parasitic zoonoses are further explored in a case series by Prof. John Frean of the South African National Institute for Communicable Diseases and the University of the Witwatersrand, describing five human cases of gnathostomiasis acquired by travelers to Botswana [5]. Travel-acquired gnathostomiasis is further explored in a report describing another case in a traveler, by Dr. Sarah Sapp and colleagues from the Centers for Disease Control and Prevention and the New York City Health and Hospitals Corporation. This report describes the discovery of a sub-adult *Gnathostoma spinigerum*, which emerged from the skin of a traveler from Bangladesh in temporal association with the patient receiving praziquantel treatment for suspected schistosomiasis [6]. 

Chronic parasitic infections in military veterans was another area of research to which Prof. Goldsmid provided several important contributions. This theme is admirably addressed by Assoc. Prof. Wayne Melrose and Prof. Peter Leggat, who describe and discuss the modern implications of the outbreak of lymphatic filariasis (LF) in United States armed forces deployed to the Pacific Islands during World War 2. Not only do these authors provide a comprehensive summary of this outbreak and its long term impact on those affected veterans, they also use it as an example of why care must be taken to avoid resurgences of LF in the many Pacific islands where elimination has recently been achieved [7]. 

The diagnosis of parasitic diseases was a major focus of Prof. Goldsmid’s research and this theme is admirably addressed in the work by Dr. Inega Gow, supervised by Dr. Damien Stark, describing the development and validation of a novel real-time PCR assay for the detection, identification and semi-quantitation of *Leishmania* spp. causing cutaneous leishmaniasis [8]. Recognizing Prof. Goldsmid’s prodigious knowledge and capability in the diagnosis of difficult and exotic parasitic zoonoses, Dr. Harsha Sheorey contributes an engaging and informative summary of the use of e-diagnosis for the identification of diagnostically challenging parasitic infections and its application to modern cases in medical parasitology [9].

This collection of papers are a testament to the many diverse topics and special areas that Prof. Goldsmid worked on in his distinguished career. The wide range of topics, such as medical parasitology, marsupial diseases in Tasmania, infections in travelers and military veterans, cultural aspects of medical practice, rare zoonotic infections in both Africa and Australia, and the diagnosis of parasitic diseases, reflect the diversity and depth of Prof. Goldsmid’s work. Prof. Goldsmid’s contributions define him as a great parasitologist, a great Tasmanian, and a great Australian, whose career and life’s work has had a substantial impact in the field of parasitology and zoonoses in Australia and Africa. We hope that his prodigious work, deep care for the health and lives of others, and inspiration of younger parasitologists, has been appropriately recognized and reflected in a small part by the high quality and wide variety of papers published in this special edition in his honor.
